# Are Atrial Fibrillation Risk Loci Universally Applicable? Insights from Whole-Genome Sequencing in a Polish Population

**DOI:** 10.3390/medsci14010155

**Published:** 2026-03-21

**Authors:** Michał Wasiak, Mateusz Sypniewski, Paula Dobosz, Maria Stępień, Anna Michalska-Foryszewska, Patryk Rzońca, Zbigniew J. Król

**Affiliations:** 1 Department of Pharmacology, Faculty of Medical Science, Cardinal Stefan Wyszynski University in Warsaw, Dewajtis 5 St., 01-815 Warsaw, Poland; 2 Department of Genetics and Animal Breeding, Poznan University of Life Sciences, 60-637 Poznan, Poland; 3 Department of Pathomorphology and Clinical Immunology, Poznan University of Medical Sciences, 60-812 Poznan, Poland; 4 Department of Infectious Diseases, Doctoral School, Medical University of Lublin, 20-059 Lublin, Poland; 5 National Medical Institute of the Ministry of the Interior and Administration, Wołoska 137, 02-507 Warsaw, Poland; 6 Department of Human Anatomy, Faculty of Health Sciences, Medical University of Warsaw, 02-091 Warsaw, Poland; 7 Department of Public Health, Wroclaw Medical University, 51-618 Wrocław, Poland

**Keywords:** atrial fibrillation, cardiovascular diseases, genetic variants, GWAS, arrhythmia risk factors

## Abstract

**Background:** Atrial fibrillation (AF) is the most common sustained cardiac arrhythmia worldwide and has a substantial genetic component. Genome-wide association studies (GWASs) have identified more than 100 susceptibility loci; however, replication across populations remains variable, suggesting potential population-specific differences in the genetic determinants of AF. To date, no whole-genome sequencing (WGS)-based study has evaluated AF susceptibility in a Polish population. **Methods:** We performed WGS (mean coverage 35×) in 233 unrelated individuals recruited within the Thousand Polish Genomes Project, including 56 patients with non-valvular AF and 177 controls without AF. After quality control and linkage disequilibrium pruning within a cardiovascular gene panel, 19,395 variants were analyzed. Association testing was performed using logistic regression adjusted for age and sex, applying both false discovery rate and Bonferroni correction thresholds. **Results:** No variants reached statistical significance for association with AF after correction for multiple evaluation. Previously reported susceptibility loci were not replicated in this cohort. Age was strongly associated with AF risk, whereas sex showed no significant effect. Given the relatively modest sample size, the study was primarily powered to detect variants with moderate or large effect sizes; smaller genetic effects reported in large GWASs may remain undetected. **Conclusions:** This pilot WGS-based study provides an initial exploration of AF-associated genetic variation in a Polish population. The absence of significant associations likely reflects the importance of further investigation in larger and well-characterized Central–Eastern European cohorts before genetic risk stratification approaches can be broadly applied across populations.

## 1. Introduction

Atrial fibrillation (AF) is the most common sustained arrhythmia in adults and represents a major contributor to cardiovascular morbidity and mortality, primarily through an increased risk of thromboembolic stroke associated with left atrial thrombus formation [1]. Globally, AF affected over 52 million individuals in 2021, with incidence rates of approximately 52 cases per 100,000. Its prevalence increases markedly with age, rising from 2–4% in the general adult population to 10–17% among individuals older than 80 years [2].

Age is the paramount risk factor for AF, and the ageing of populations worldwide is expected to substantially increase its burden. In the United States, the number of individuals with AF is projected to reach 6–12 million by mid-century, while in Europe incident cases are also expected to rise significantly in the coming decades [2]. In Poland, AF prevalence has been reported to reach approximately 19.2% among individuals aged ≥65 years. Comorbidities like hypertension, obesity, coronary disease, valvular pathology, sleep apnoea, and kidney disease are known to increase the risk of AF [3].

Although AF is most commonly associated with structural heart disease, cases occurring in structurally normal hearts often show familial clustering, with an elevated risk among first-degree relatives and increased concordance in monozygotic twins supporting the heritability of the condition [4,5,6]. To date, rare pathogenic variants have been identified in numerous genes involved in cardiac electrophysiology and structural integrity, including ion channels, sarcomeric proteins, transcription factors, and gap junction proteins. In parallel, genome-wide association studies (GWASs) have identified more than 150 common susceptibility loci associated with AF risk, including well-established regions near developmental regulators such as *PITX2* on chromosome 4q25 [7,8,9]. Recent large multi-ancestry GWASs involving more than one million individuals have continued to expand the number of loci associated with AF susceptibility [10].

Despite these advances, the genetic architecture of AF remains incompletely characterized in many populations, including those from Central and Eastern Europe. In particular, population-specific data derived from sequencing-based approaches remain limited.

### Study Focus

In this study, we analyzed whole-genome sequencing data from individuals included in the Thousand Polish Genomes Project to explore genetic variation potentially associated with AF in a Polish cohort. Using a targeted analysis of cardiovascular-related genes, we evaluated common variants in patients with non-valvular AF and controls without documented AF. This analysis aimed to assess whether variants previously implicated in AF susceptibility are detectable in this population and to provide initial data for future studies in Central–Eastern European cohorts.

## 2. Results

The study included 233 unrelated individuals recruited under the frame of the Thousand Polish Genomes Project, a nationwide effort to define the genetic variability of the Polish population. The study population consisted of 56 patients diagnosed with non-valvular AF and 177 controls without the arrhythmia. Several comorbidities were more prevalent in the AF group. In particular, hypertension, diabetes mellitus, coronary heart disease, heart failure, prior stroke/TIA, hyperlipidemia, and smoking history were observed more frequently among patients with AF. Detailed baseline clinical characteristics of both groups are presented in Table 1.

**Figure 1:**
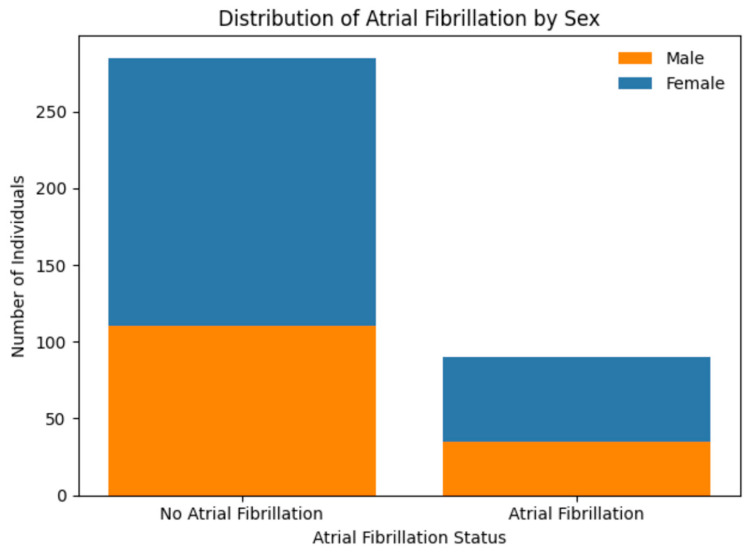
Distribution of males and females among AF and control groups.

As illustrated in Figure 1, the distribution of sexes was balanced between groups, with a slight male predominance in both the AF (34 males, 21 females) and control (109 males, 68 females) cohorts. Logistic regression models excluding SNP effects demonstrated that sex did not exert a statistically significant independent effect on AF risk in this specific cohort (*p* > 0.05), which differs from some previous reports but aligning with findings that sex differences in AF prevalence may be mediated by differential exposure to risk factors across populations.

**Figure 2:**
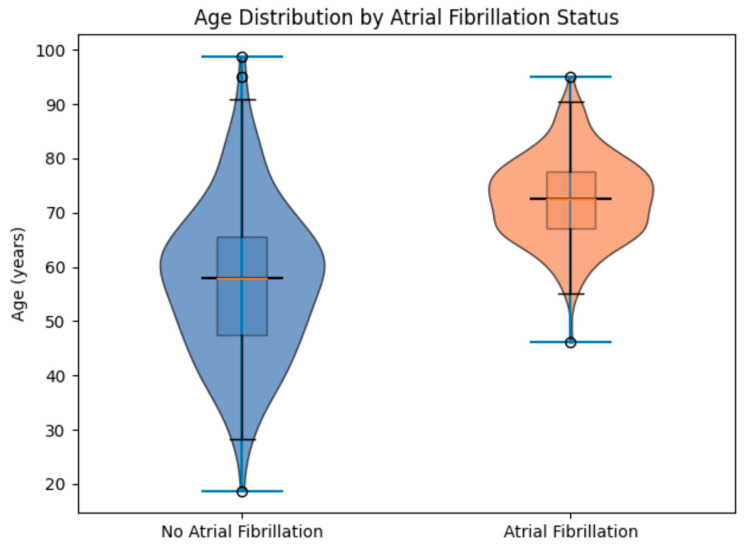
The distribution of age at hospital admission in the AF and control groups. The *t*-test for age was highly significant (*p* ≈ 6 × 10^−16^).

Conversely, age at hospital admission was a strong and statistically significant predictor, as shown in the violin plots in Figure 2. The AF group was substantially older (mean 73.1 years, range 51–93) than the control group (mean 57.7 years, range 19–99), reflecting the age-dependent nature of AF substrate progression. Statistical modelling indicated that for every additional year of age, the odds of an AF diagnosis increased by a factor of 1.085, equivalent to an 8.5% annual escalation in risk.

Laboratory findings revealed significantly lower red blood cell (RBC) counts and borderline lower hemoglobin levels in AF patients. These observations are unlikely to represent primary genetic hematological disorders; rather, they are consistent with the physiological consequences of chronic anticoagulation therapy, which is the standard of care for AF to mitigate thromboembolic risks. Clinically, these findings highlight the intersection of comorbid management and baseline laboratory profiles in ageing AF populations [11].

Apart from differences in age and hematological parameters, the AF group had a significantly higher prevalence of several cardiovascular comorbidities compared with the control group, including hypertension, diabetes mellitus, coronary heart disease, heart failure, prior stroke/TIA, hyperlipidemia, and smoking history.

The genomic analysis focused on cardiovascular-related genes identified from whole-genome sequencing data. Sequencing was performed on the Illumina NovaSeq 6000 platform with 150 bp paired-end reads, achieving a mean depth of coverage of 35.26× across the cohort. Following quality control filtering, linkage disequilibrium (LD) pruning was applied (50 kb window, r^2^ > 0.5) to select representative variants within regional haplotype blocks. After these procedures, a total of 19,395 single-nucleotide polymorphisms (SNPs) and short insertions/deletions were retained for association analysis.

The association between genetic variants and AF was evaluated using logistic regression. The log-odds of AF were modelled as follows:

logit(P) = ln(P/(1 − P)) = β0 + β1G + β2Age + β3Sex

where P denotes the probability of AF, G represents the genotype coded under an additive genetic model (0, 1, or 2 copies of the effect allele), and Age and Sex were included as covariates. The reference category corresponded to individuals homozygous for the reference allele.

**Figure 3:**
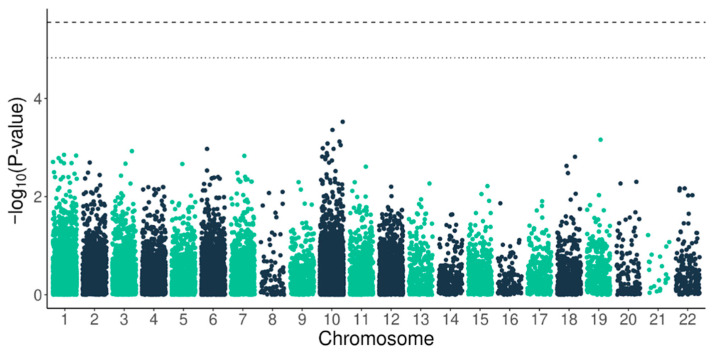
A Manhattan plot for variants associated with AF. The upper dashed line represents the Bonferroni-corrected significance threshold (*p* = 0.05), while the lower indicates the suggestive significance threshold.

The Manhattan plot (Figure 3) illustrates the distribution of association signals across the analyzed variants and confirms the absence of genome-wide significant associations in this cohort. Variants previously reported in large GWASs were not replicated at statistically significant levels in this dataset. However, given the relatively modest sample size, the present study was primarily powered to detect variants with moderate to large effect sizes, and smaller effects reported in large population studies may remain undetected.

Variants located within or near the *NEBL* (Nebulette) gene did not show statistically significant association with AF in this cohort. Although NEBL has been previously discussed in the context of cardiac structural proteins and atrial remodelling, the present data do not support a detectable association between common variants in this gene and AF susceptibility in the analyzed population.

## 3. Discussion

This study represents, to our knowledge, the first whole-genome sequencing (WGS)-based analysis exploring genetic variation potentially associated with AF in a Polish cohort. Using high-coverage sequencing data derived from the Thousand Polish Genomes Project, we evaluated the association between AF and common genetic variants within a panel of cardiovascular-related genes [12].

Despite comprehensive genomic coverage and high sequencing depth, no variants reached statistical significance after correction for multiple testing. Previously reported susceptibility loci identified in large GWAS meta-analyses were also not replicated in this dataset [10,13]. However, this finding should be interpreted cautiously given the relatively modest cohort size of 56 AF cases and 177 controls. Large population studies such as the AFGen Consortium or UK Biobank include thousands of cases and are therefore powered to detect variants with small effect sizes, which represent the majority of genetic associations reported for AF [10]. In contrast, the present study was primarily powered to detect variants with moderate or large effects.

Large-scale meta-analyses, such as the landmark study by Roselli et al. (2020), have identified over 138 loci associated with AF susceptibility, including well-established regions near *PITX2* and *ZFHX3* [10]. These discoveries highlight the complex polygenic architecture of AF. Nevertheless, replication of individual loci may vary between populations due to differences in allele frequency, linkage disequilibrium structure, environmental modifiers, or statistical power [14]. Therefore, the absence of significant associations in the current study should not be interpreted as evidence that previously reported loci are absent in the Polish population, but rather that small-effect variants identified in large GWASs may remain undetected in smaller cohorts.

Population-specific differences in the genetic architecture of AF have been reported in several studies. For example, Low et al. (2017) identified several loci in a Japanese cohort, including variants near *KCND3*, *HAND2*, and *NEBL*, that demonstrated ancestry-dependent effects and were not consistently replicated across other populations [15]. Such observations illustrate the complexity of cross-population replication in complex polygenic traits and highlight the importance of evaluating genetic associations in diverse populations [14].

In the present study, none of the 19,395 analyzed cardiovascular-related variants showed statistically significant association with AF after correction for multiple testing [12]. Age remained the strongest independent predictor of AF risk in the multivariable logistic regression model (OR 1.085 per year). This observation is consistent with extensive epidemiological evidence demonstrating that ageing is the dominant clinical determinant of AF development [16]. Although traditional cardiovascular risk factors such as hypertension, diabetes mellitus, coronary artery disease, and heart failure were more prevalent among patients with AF in the baseline analysis, their associations were attenuated after adjustment in the multivariable model. In Polish seniors ≥65, AF prevalence surges to 19.2%, with paroxysmal forms leading the rhythm rebellion [16].

The Polish results suggest that Slavic populations may represent a third distinct genetic architecture that differs significantly from both Western Europeans and East Asians [12,17]. Furthermore, the lack of a significant association for any of the 19,395 cardiovascular-relevant variants in the current study indicates a null result for established markers, with the epidemiological signal instead being dominated by age [10]. This underscores the critical need for regional biobanks, as the “one-size-fits-all” approach to genomic risk prediction is likely to exacerbate health disparities by providing less accurate risk stratification for underrepresented populations [17]. The above-mentioned studies have been summarized and are compared in Table 2.

The investigation of the *NEBL* gene in this Polish cohort provides insight into the potential role of structural genes in AF etiology. However, our data do not support a direct association between common *NEBL* variants and AF susceptibility in this population [12]. NEBL encodes nebulette for the Z-disc protein that interacts with actin and α-actinin and plays an important role in maintaining sarcomeric integrity in cardiomyocytes [11,12]. Pathogenic mutations in this gene have previously been associated with dilated, hypertrophic, and left ventricular non-compaction cardiomyopathies, conditions characterized by structural myocardial abnormalities and impaired contractile function [19].

Recent studies have suggested that genes primarily involved in cardiomyopathy may also contribute to AF risk, even in the absence of overt heart failure, likely through mechanisms related to atrial structural remodelling and fibrosis [20]. In this context, NEBL may represent a structural modifier that contributes to the development of atrial cardiomyopathy, a substrate characterized by atrial hypertrophy, fibrosis, and increased susceptibility to re-entrant arrhythmias [21]. Although our results do not demonstrate a significant association between common *NEBL* variants and AF, rare or functionally disruptive variants in this gene may still contribute to AF susceptibility in selected individuals.

The analysis of variants within the *NEBL* gene did not demonstrate statistically significant association with AF susceptibility in this population. Although structural genes involved in cardiomyocyte integrity have been discussed in the context of atrial remodelling and cardiomyopathy, the present data do not support a direct role for common *NEBL* variants as primary susceptibility factors for AF in this cohort.

Overall, these findings highlight the importance of evaluating genetic associations across diverse populations and underscore the need for larger sequencing-based studies in Central and Eastern European cohorts. The present work represents an exploratory step toward better characterization of AF genetics in underrepresented populations and provides a basis for future multicentre investigations incorporating larger cohorts and integrated genomic analyses.

Ultimately, the strong association between age and AF risk (OR 1.085 per year) remains the dominant epidemiological signal, reinforcing the view that in contemporary clinical cohorts, structural and environmental remodelling driven by ageing often eclipses the subtle signals of common genetic variants [12]. Power favours bold effects in our n = 56 setup, blind to subtle OR < 1.2 whispers needing thousands for detection [22]. Bonferroni bites conservatively, masking nominal flirts, while age skew (*p*~6 × 10^−16^) confounds lone AF signals [12]. Yet WGS’s comprehensive gaze, from coding to rare non-coding, outshines arrays, nailing nulls precisely [10,23].

Recent polygenic risk score (PRS) audits reveal the following: high scores double AF odds, but ancestry tweaks are vital for accuracy [24,25,26]. The lack of replication in this study highlights significant challenges for the clinical implementation of PRSs in diverse populations and emphasizes the critical importance of external validity [17,26]. The “transportability” of causal inference, or the ability to apply effect estimates from a source population to a different target population, is compromised when the distribution of genetic ancestry, mediators, or effect modifiers differs across groups. Consequently, causal inferences and AF risk stratification models derived from Western European or East Asian cohorts may not remain valid or directly applicable to Central–Eastern European populations without rigorous local validation. For example, the *PITX2* and *ZFHX3* loci, which serve as the cornerstones for most AF polygenic risk models, showed no evidence of significance in this Polish whole-genome sequencing (WGS) cohort [10,12,17]. If these primary drivers are not transportable, the cumulative predictive accuracy of current PRSs will be markedly lower for Polish patients, potentially leading to suboptimal screening and prevention strategies.

This study also reinforces the emerging “atrial-first phenotype” hypothesis, which posits that genetic susceptibility to atrial cardiomyopathy and structural remodelling may be a more potent driver of AF in some populations than the electrical triggers identified in broad GWAS [27]. Identifying rare pathogenic variants in genes like *TTN or LMNA* in Polish patients may therefore offer a more reliable path toward precision management than the application of common SNP-based risk scores [5,17].

The interpretation of our results must account for critical statistical constraints, primarily focused on the rigorous maintenance of scientific objectivity [22]. Statistical power represents the most significant limitation; given the modest sample size of n = 56 AF cases, this study was powered primarily to detect genetic variants of moderate-to-large effect sizes. However, large-scale meta-analyses consistently demonstrate that the majority of AF-associated variants discovered to date have small effect sizes (OR < 1.2), which would require thousands of cases to detect reliably; thus, our lack of association does not exclude the existence of common small-effect loci [22]. Willer et al. (2010) emphasized that meta-analysis remains the gold standard for maximizing power to detect such subtle effects for common traits [22].

Furthermore, the application of the Bonferroni correction for multiple testing is extremely conservative for a pilot study, potentially obscuring variants that reached nominal significance but failed the adjusted threshold [10,12,22]. Residual confounding may also persist due to the significant age imbalance between the AF and control groups (*p* ≈ 6 × 10^−16^), which may limit the detection of age-interacting genetic signals or bias the results toward factors manifesting in younger “lone AF” patients [12].

Despite these constraints, the strength of the methodology lies in the use of high-depth whole-genome sequencing (WGS) rather than traditional array-based genotyping [12]. While traditional GWASs capture mostly non-coding variants through imputation, WGS provides a comprehensive catalogue of both coding and non-coding variations, including rare mutations often missing from population-wide panels [10,23]. By focusing on a targeted cardiovascular gene panel within the WGS data, this study maximized clinical relevance, ensuring the null result accurately reflects the absence of large-effect variants within the Polish population [12].

Within our cohort, age emerged as the dominant independent determinant of AF, reinforcing the concept that progressive atrial substrate remodelling frequently eclipses the modest effect sizes of common genetic variants, particularly in moderately powered population studies. These data underscore a fundamental principle: demographic and structural determinants may outweigh polygenic susceptibility when disease expression reflects decades of cumulative structural remodelling.

These findings further refine the role of *NEBL*. Rather than functioning as a primary susceptibility gene in the Polish population, *NEBL* appears more plausibly positioned as a structural contributor whose influence on AF is indirectly mediated through atrial cardiomyopathy and structural remodelling processes. Distinguishing between genes that initiate electrical instability and those that shape the structural substrate is essential for mechanistic clarity and for preventing misinterpretation of association signals as evidence of direct causal effects. Most importantly, this work underscores a broader imperative for precision cardiology. Validation of genetic risk loci across distinct European subpopulations is increasingly necessary. It is a prerequisite for responsible clinical translation. Before polygenic risk scores can inform screening or preventive strategies for millions of individuals in Central and Eastern Europe, population-specific calibration through regional meta-analyses valuable.

This study represents a foundational pilot analysis. It marks a transition from passive adoption of global discovery signals toward the development of regionally grounded genomic evidence. By defining both the loci that replicate and those that do not, we take a necessary step toward bridging the gap between international genomic research and population-specific cardiovascular risk assessment and care.

## 4. Conclusions

This study provides a whole-genome sequencing-based framework for investigating the genetic background of AF in a Polish population. The results contribute to the characterization of AF genetic architecture in Central and Eastern European populations and provide a reference point for future population-specific studies. The lack of replication of several previously reported susceptibility loci, including variants attributed to *NEBL*, suggests that the genetic determinants of AF may differ across populations. These findings highlight the potential population specificity of genetic risk architecture and underscore the need for further large-scale genomic studies in underrepresented European populations.

## 5. Materials and Methods

### 5.1. Sample Collection

Blood samples were collected from 232 unrelated individuals across Poland between April 2020 and April 2021, recruited by the Central Clinical Hospital of the Ministry of Interior and Administration in Warsaw, within the frame of the 1000 Polish Genome Project (https://doi.org/10.3390/ijms23094532, accessed: 1 June 2022) [12]. For all individuals, basic clinical data, including age, gender, body weight and basic laboratory results, were collected. Only individuals without severe heart valve disease, end-stage heart failure and cancer (till the moment of sample collection) were qualified for this study. Within this cohort, 56 patients were diagnosed with AF (based on their medical records, ECG and confirmed diagnosis). A total of 177 patients without AF were treated as a control group. This group consisted of patients (i) without congenital heart defects nor history of heart surgery or moderate/ severe heart valve disease; (ii) with no AF records medical history and/or ECG and/or Holter ECG during hospitalization. AF diagnosis criteria were coherent with the latest American Heart Association (AHA), American College of Cardiology (ACC) and Heart Rhythm Society (HRS) guidelines [28,29]. All types of AF (paroxysmal, long lasting, permanent) were included. Regarding ethnicity, our cohort consists of patients representing the Polish population, which can be considered genetically homogenous based on studies that define the mtDNA variability of the Polish population and visualize genetic relations between Poles [30].

### 5.2. Total Quality Management

The project was carried out in accordance with the Total Quality Management (TQM) methodology, which ensures the quality of results and analyses the risk and possible difficulties. TQM requires defining all critical points of the procedures: reference ranges for collected biological material, its preparation, isolation, DNA concentration and quality, genomic sequencing, and quality control of the data. The legal and ethical transparency of the entire project was ensured, including the confidentiality, integrity, and impartiality of the data.

### 5.3. Whole-Genome Sequencing (WGS)

The whole genomes of 233 unrelated participants were sequenced in this study [more information in our previous report: [31]). A total of 4 mL of K-EDTA peripheral blood from participants was collected according to a standardized Quality Management System protocol. Genomic DNA was isolated from the peripheral blood leukocytes using a QIAamp DNA Blood Mini Kit, Blood/Cell DNA Mini Kit (Syngen, Wrocław, Poland) and Xpure Blood Kit (A&A Biotechnology, Gdańsk, Poland) according to the manufacturers’ protocols. The concentration and purity of isolated DNA were measured using the NanoDropTM spectrophotometer, and the quality of the DNA was evaluated using gel electrophoresis. The sequencing library was prepared by Macrogen Europe (Amsterdam, The Netherlands) using TruSeq DNA PCR-free kit (Illumina Inc., San Diego, CA, USA) and 550 bp inserts. Quality of DNA libraries was measured using 2100 Bioanalyzer, Agilent Technologies (Santa Clar, CA, USA). Whole-genome sequencing (WGS) was performed on the Illumina NovaSeq 6000 platform using 150 bp paired-end reads, yielding a mean depth of coverage of 35.26X in the cohort.

### 5.4. Association Study

Whole-genome sequencing identified 38,296,203 single-nucleotide polymorphisms (SNPs) and short insertions/deletions. To focus the analysis on variants with potential cardiovascular relevance, we restricted the association analysis to a predefined panel of 203 genes previously implicated in cardiovascular diseases. This panel contained 274,921 SNPs and short indels.

To reduce redundancy due to linkage disequilibrium (LD), we applied LD pruning to select representative variants (tag variants) within haplotype blocks. LD pruning was performed using a 50 kb sliding window and an r^2^ threshold of 0.5, where r^2^ represents the squared correlation coefficient between alleles at two loci [32]. This procedure removes highly correlated variants while retaining representative markers for regional haplotype structure. After quality control filtering and LD pruning, a total of 19,395 SNPs and short indels remained for association analysis.

Genetic association testing was performed using logistic regression implemented in PLINK v1.9 [33]. AF status was modelled as the dependent variable, with age and sex included as covariates. Genotypes were coded under an additive genetic model (0, 1, or 2 copies of the effect allele) [34].

For each variant, odds ratios (ORs) and corresponding standard errors were estimated. Two statistical thresholds were applied to account for multiple testing:

(1) The Benjamini–Hochberg false discovery rate (FDR) < 0.1. (2) Bonferroni correction for the total number of tested variants.

### 5.5. Statistical Analysis

Statistical analyses were performed using IBM SPSS Statistics (28 (released in 2021) IBM Corp., Armonk, NY, USA). Continuous variables are presented as the mean ± standard deviation (SD), whereas categorical variables are expressed as counts and percentages. Differences between groups were assessed using Student’s *t*-test for continuous variables and the chi-square test for categorical variables.

To identify clinical factors associated with AF, logistic regression analysis was performed. The presence of AF was used as the dependent variable. Independent variables included age, sex, hypertension, diabetes mellitus, coronary heart disease, heart failure, prior stroke or transient ischemic attack, chronic kidney disease, hyperlipidemia, and smoking status. Odds ratios (ORs) with 95% confidence intervals (CIs) were calculated.

A two-sided *p*-value < 0.05 was considered statistically significant.

### 5.6. Limitations of Study

A limitation of the present study is the relatively modest number of AF cases (n = 56), which limits the statistical power to detect common variants with small effect sizes. Large-scale GWAS meta-analyses demonstrate that most AF-associated loci result in modest risk increases (odds ratios typically ranging from 1.05 to 1.20). The present study was primarily powered to detect variants with moderate or large effect sizes, while smaller effects may remain undetected in this cohort. Therefore, the lack of replication of previously reported loci should not be interpreted as evidence of the absence of an effect but might be the result of limited statistical power in a pilot sequencing dataset.

## Figures and Tables

**Table 1:** Baseline characteristics of investigated groups.

Variable	AF (n = 56)	Control (n = 177)	*p*-Value
Age, years (mean ± SD)	73.8 ± 10.1	58.2 ± 17.4	<0.001
Male sex, n (%)	40 (71.4)	110 (62.1)	0.27
Hypertension, n (%)	40 (71.4)	66 (37.3)	<0.001
Diabetes mellitus, n (%)	17 (30.4)	30 (16.9)	0.047
Coronary heart disease, n (%)	22 (39.3)	13 (7.3)	<0.001
Heart failure, n (%)	17 (30.4)	13 (7.3)	<0.001
Prior stroke/TIA, n (%)	9 (16.1)	5 (2.8)	<0.001
Chronic kidney disease, n (%)	8 (14.3)	10 (5.6)	0.068
Hyperlipidemia, n (%)	23 (41.1)	11 (6.2)	<0.001
Smoking, n (%)	12 (21.4)	13 (7.3)	0.006
Red blood cell (RBC) [mln/µL] mean ± SD	3.81 ± 0.83	4.20 ± 0.74	0.0029
Hemoglobin [g/dL] mean ± SD	11.62 ± 2.49	12.67 ± 2.13	0.0064

**Table 2:** The most important studies involving the genetics behind AF.

Study	Population	Identified/Targeted Loci	Source
Roselli et al. 2020	Multi-ethnic/European	138 loci (e.g., *PITX2*, *ZFHX3*)	[10]
Low et al. 2017	Japanese	*NEBL*, *KCND3*, *HAND2*	[15]
Gudbjartsson 2007	Icelandic	4q25 (*PITX2*)	[8]
Thousand Polish Genomes	Polish	19,395 cardiovascular variants	[12]
Yuan et al. 2025	Cross-population	Proteomics-enhanced loci	[18]

## Data Availability

The dataset is freely available for researchers at https://doi.org/10.3390/ijms23094532 (accessed on 15 July 2022) or via reasonable request from the corresponding author due to privacy restrictions (https://www.mdpi.com/1422-0067/23/9/4532 (accessed on 15 July 2022)).
